# Experiences of physiotherapists regarding a standard set of measurement instruments to improve quality of care for patients with chronic obstructive pulmonary disease: a mixed methods study

**DOI:** 10.1186/s41687-022-00487-2

**Published:** 2022-07-19

**Authors:** Arie C. Verburg, Jessica Zincken, Henri Kiers, Simone A. van Dulmen, Philip J. van der Wees

**Affiliations:** 1 Radboud University Medical Center, Radboud Institute for Health Sciences, IQ Healthcare, PO Box 9101, 6500 HB Nijmegen, The Netherlands; 2 Institute of Human Movement Studies, Utrecht University of Applied Sciences, Utrecht, The Netherlands; 3 Association for Quality in Physical Therapy (SKF), Zwolle, The Netherlands

**Keywords:** COPD, Measurement instruments, Standard set, Treatment evaluation, Physiotherapy, Quality improvement

## Abstract

**Rationale:**

The quality of physiotherapy care for patients with chronic obstructive pulmonary disease (COPD) can be improved by comparing outcomes of care in practice.

**Aim:**

To evaluate the experiences of physiotherapists implementing a standard set of measurement instruments to measure outcomes and improve the quality of care for patients with COPD.

**Methods:**

This sequential explanatory mixed methods study was performed in two parts. In the quantitative part, a survey of 199 physiotherapists was conducted to evaluate their attitudes and knowledge, as well as the influence of contextual factors (i.e., practice policy and support from colleagues), in the implementation of the standard measurement set. In the qualitative part, 11 physiotherapists participated in individual interviews to elucidate their experiences using a thematical framework.

**Results:**

The survey showed that, on average, 68.4% of the physiotherapists reported having a positive attitude about using the standard set, 85.0% felt they had sufficient knowledge of the measurement instruments, and 84.7% felt supported by practice policy and colleagues. In total, 80.3% of physiotherapists thought the standard set had added value in clinical practice, and 90.3% indicated that the measurement instruments can be valuable for evaluating treatment outcomes. The physiotherapists mentioned several barriers, such as lack of time and the unavailability of the entire standard set of measurement instruments in their practice. Moreover, the physiotherapists indicated that the measurement instruments have added value in providing transparency to policymakers through the anonymized publication of outcomes.

**Conclusion:**

Physiotherapists support the use of the standard set of measurement instruments to improve the quality of physiotherapy treatment for patients with COPD.

**Supplementary Information:**

The online version contains supplementary material available at 10.1186/s41687-022-00487-2.

## Introduction

Chronic obstructive pulmonary disease (COPD) is a serious public health problem. This progressive disease affects the lungs, causing dyspnoea with exertion in particular, which has a negative effect on quality of life [1]. Physiotherapy improves the quality of life of patients with COPD by increasing the physical capacity and decreasing breathlessness [2]; thus, high-quality physiotherapy care for these patients is of high importance for achieving optimal treatment results. In recent years, routinely collected real-world data from electronic health records have become available from national data registries in the Netherlands. These data offer the opportunity to use patient outcomes in the interaction between the physiotherapist and the patient (e.g., in goal setting and shared decision-making), and to improve the quality of care by learning from aggregated outcomes within and between practices [3–5]. Furthermore, routinely collected data may be used for external transparency, such as public reporting or pay-for-performance initiatives [6]. It is important that valid outcomes and measurement instruments are selected, tested for their use in quality improvement, and validated by end users [7, 8].

Previous research investigated the barriers to and facilitators of physiotherapists using measurement instruments, revealing that they were not being routinely used [9–12]. It was found that, although physiotherapists had a positive attitude towards the use of measurement instruments, they were not always sure which should be used for which patient. They indicated that a standard set of measurement instruments is needed, including instructions for their use and interpretation [7]. The present lack of standardization in outcome measurements has meant that physiotherapy care approaches cannot be properly compared and evaluated [9].

A standard set of measurement instruments for Dutch physiotherapist practice, including patient-reported outcome measures (PROMs) and physical performance tests, was developed for patients with COPD and registered on the COMET website [13, 14]. Physiotherapists can use this set for diagnostic purposes, goalsetting, and evaluating the outcomes of physiotherapy treatments for patients with COPD; however, it is unclear whether this standard set overcomes the described barriers for the successful implementation of routine data collection and the use of outcomes data to stimulate quality improvement.

Thus, the objectives of this study were (1) to evaluate the implementation of the set of measurement instruments for patients with COPD undergoing physiotherapy, and (2) to explore the perceptions of physiotherapists regarding the use of the set for goalsetting, quality improvement, and external transparency.

## Methods

### Study design

A mixed methods approach with an explanatory sequential design was used by means of a survey and interviews with Dutch primary care physiotherapists. The standard set of measurement instruments was developed in a previous study [13], and included measures of the process and outcomes of physiotherapy care. Details of the set are available in Additional file 1. The set was implemented in a two-year time frame (January 2018 to December 2019) in 156 primary care practices, involving 295 physiotherapists [15]. Twice a year, the participating practices received a report comparing their own collected data with benchmark data, presented in caterpillars plots [15].

**Fig. 1:**
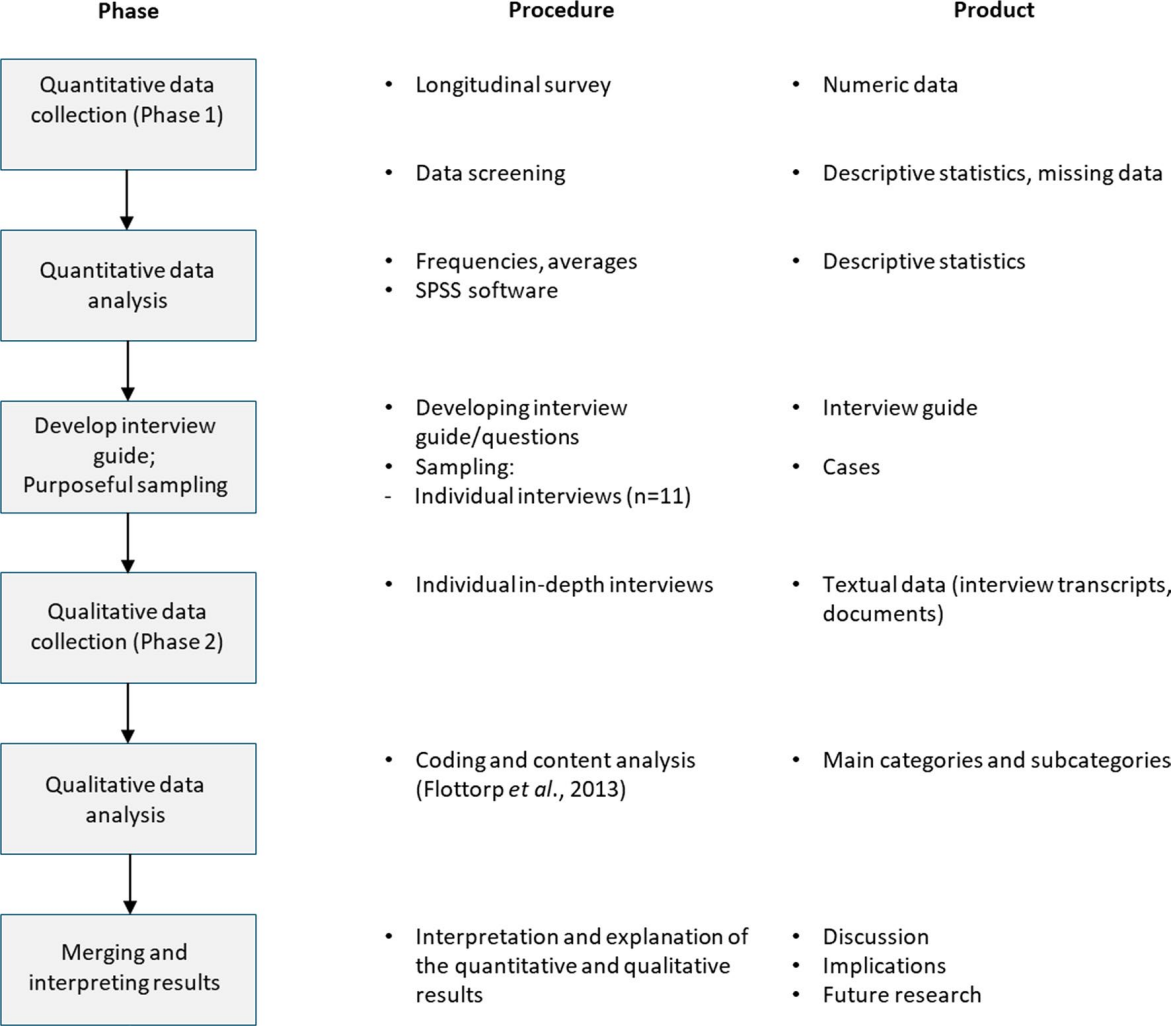
Diagram of the study. A sequential explanatory mixed method design

The present study included two phases (see Fig. 1). During the first phase, quantitative data from a survey of physiotherapists were analysed to evaluate their attitudes, knowledge, and the influence of contextual factors (i.e., practice policy and support from colleagues) in the use of the standard set for improving the physiotherapy treatments for patients with COPD. In the second phase, in-depth interviews were held with physiotherapists to gain a better understanding of their experiences of implementing the standard set of measurement instruments. The survey was executed from April–June 2018, while the interviews were conducted in March–June 2020.

The study protocol was approved by the Medical Ethical Committee of Radboud university medical centre (Registration #2019-5455). The informed consent of each participant was obtained.

#### Phase 1: survey

### Data collection

All physiotherapists who participated in the previous implementation study were invited to participate. A survey was sent via LimeSurvey version 2.06, with a total of three reminders. The survey was based on the previously developed ‘PROM use self-assessment questionnaire’ [3]. The questions were allocated into three domains: attitude, knowledge, and context [3]. The questionnaire asked for the (demographic) characteristics of each participant, their perceptions of the implementation of the standard set, their personal experience with the standard set, and the policy of their practice regarding the standard set. All questions were scored on a five-point Likert scale (1 = totally disagree, 5 = totally agree). Some minor changes were made to the original questionnaire because the current study used a specific standard set of measurement instruments instead of the general PROMs explored in the original article; for example, ‘I know where to find PROMs’ was changed to ‘I know where to find the measurement instruments.’

### Data analysis

Mean scores and standard deviations (SD) were calculated for each question and for the three domains. Descriptive statistics were calculated to describe the percentage of physiotherapists who agreed on the questions provided. A score of 4 (agree) or 5 (totally agree) on a question was interpreted as agreed. SPSS version 25 was used for all calculations.

#### Phase 2: interview study

The protocol for the interviews was based on the results of the survey. The questions were designed to identify the factors that potentially facilitate or hinder the use of the measurement instruments. Physiotherapists who had completed the survey in the first part of the study were eligible to participate in the interviews once their consent had been obtained. The physiotherapists were purposefully selected based on their demographic characteristics collected in the survey, including age, gender, working hours, and geographic location across the Netherlands. Data saturation was expected to be reached after 10–15 interviews. The COREQ checklist [16] was used as guidance for conducting all aspects of the qualitative research.

### Data collection

Semi-structured in-depth interviews were pilot tested and conducted via video connection and audio recorded by one researcher (JZ). The interviews began with some general open-ended questions, after which the physiotherapists were asked about their experiences with the use of measurement instruments. Finally, the physiotherapists were asked about their perspectives on the potential use of the data for quality improvement and transparency (see Additional file 2 for the interview guide).

### Data analysis

The interviews were transcribed verbatim. The transcripts were entered into Atlas.ti, a program used for analysing qualitative data, assigning codes, and allocating the codes into categories. We used a directed approach to content analysis [17]. We inductively coded the transcripts and then used an existing theoretical framework to guide higher-order clustering. Specifically, two researchers (JZ and AV) independently coded two transcripts and discussed the codes to reach a consensus. The remaining transcripts were coded by one researcher (JZ) and checked by another researcher (AV). During this process, new codes were added when needed after discussions between both researchers (JZ and AV). Based on their similarities, the codes were allocated into categories (by JZ and AV). Then we used the theoretical framework of Flottorp et al. [18] to cluster the codes to major categories and the seven domains of Flottorp: Guideline factors; Individual health professional factors; Patient factors; Professional interactions; Incentives and resources; Capacity for organizational change; and Social, political and legal factors. The theoretical framework of Flottorp facilitates the evaluation and reporting of tailored interventions. The clustering of the categories to the domains was discussed during meetings with all research members (JZ, AV, SvD, and PvdW) to reach a consensus. The research team (JZ, AV, SvD, and PvdW) also held several meetings throughout data collection to discuss and interpret the preliminary findings, to make potential amendments to the interview guide, and to identify whether data saturation had been reached.

### Trustworthiness

Both the quantitative and qualitative parts of this study are related to the validity of a mixed method design. A large sample size was used for the survey to minimize bias and possible validity threats. Interviews were held until data saturation was reached. The participants had no personal relationship with the researchers. The interview data were analysed by both JZ (a master’s student) and AV (a physiotherapist and PhD student) to strengthen trustworthiness. On several occasions during the study, all research members (JZ, AV, PvdW (a physiotherapist and professor of allied health sciences), and SvD (a physiotherapist and senior researcher)) discussed the codes, categories, and domains to reach consensus about the findings from the interviews. The research members were trained in (and most had experience in) conducting qualitative research (AV, SvD, HK (a physiotherapist and senior researcher), and PvdW). Reliability and validity were established using the four components outlined by Guba and Lincoln (1981): credibility, transferability, dependability, and confirmability [19]. The identities of the physiotherapists were considered confidential; therefore, the answers given by the physiotherapists during the interviews and in the survey were processed anonymously. Meaning that the transcripts of interviews in the current study cannot be linked to identities of participants by removing all identifiable information of the participants.

## Results

### Survey

Of the 295 physiotherapists who participated in the implementation study, a total of 199 completed the survey (response rate: 67.4%). The mean age was 42.1 years (SD 12.0), and 92 participants were male (46.2%). The participating physiotherapists comprised a representative sample in terms of age and gender when compared with the national reference data [20]. The mean number of hours worked per week among the male participants was 37.3 h (SD 6.7 h), whereas the mean working hours per week among the female participants was 28.4 h (SD 6.1 h). See Table 1 for full details.

**Table 1:** Characteristics of the physiotherapists participating in the survey

	N	Age in years (SD)	Working hours per week (SD)
All participants	199	42.1 (12.0)	32.5 (7.7)
Male (%)	92 (46)	43.6 (13.0)	37.3 (6.7)
Female (%)	107 (54)	40.8 (10.9)	28.4 (6.1)

*SD* standard deviation

The results of the survey showed that the majority of respondents had positive opinions of the use of the measurement instruments and the implementation of the standard set. Table 2 provides a complete overview of the results of the survey per item and per category. Some items might have a slightly different response rate as not all participants answered all questions. Table 2 shows that 68.4% of the physiotherapists (in total) agreed with items related to having a positive attitude (mean score 3.88), 85% (in total) agreed with the items related to having sufficient knowledge (mean score 4.06), and a total of 84.7% agreed with the items related to context (mean score 4.16). This indicates that the highest gains in the implementation of the set of measurement instruments in clinical practice could be made by changing the attitude of the physiotherapists regarding the use of the standard set in daily practice. Of the physiotherapists who completed the survey, 91.7% agreed that the measurement instruments are useful in the evaluation of the treatment; 23.8% agreed that they would like to use the measurement instruments more often in clinical practice.

**Table 2:** Results of the survey on attitude, knowledge, and context

	% (in total) who agree^‡^	Mean (SD)	Min–max^§^
Attitude	68.4	3.88 (0.86)	1–5
Using the measurement instruments helps me formulate a physiotherapeutic diagnosis	69.4	3.74 (0.86)	1–5
The measurement instruments are useful in the evaluation of the treatment	91.7	4.18 (0.69)	1–5
The measurement instruments have a positive influence on the quality of physiotherapy healthcare	74.1	3.83 (0.83)	1–5
It is important to register patient experiences objectively with the measurement instruments	87.6	4.07 (0.96)	1–5
Using the measurement instruments in clinical practice does takes too much time^†^	51.7	4.60 (1.00)	1–5
I would like to use the measurement instruments more often in clinical practice	23.8	2.82 (0.96)	1–5
I have experienced the added value of the measurement instruments in clinical practice	80.8	3.96 (0.72)	1–5
Knowledge	85.0	4.06 (0.72)	1–5
I know where to find the measurement instruments	93.3	4.31 (0.76)	1–5
I am capable of using the measurement instruments with my patients	93.7	4.31 (0.71)	1–5
I am able to interpret the results of the measurement instruments	91.7	4.19 (0.69)	1–5
Using the measurement instruments does not affect my professional authority to make my own decisions	79.3	3.84 (0.79)	1–5
All patient needs can be registered in the measurement instruments	50.2	3.40 (0.77)	1–5
I am able to use the measurement instruments within physiotherapeutic methodical action	93.8	4.17 (0.62)	1–5
I use the measurement instruments in daily practice	93.3	4.26 (0.68)	1–5
Context	84.7	4.16 (0.74)	1–5
The use of the set measurement instruments fits with how I am used to working	71.0	3.70 (0.74)	2–5
The measurement instruments are available in my practice	92.4	4.41 (0.67)	1–5
In our practice, we have made arrangements for how to use the measurement instruments	84.4	4.09 (0.85)	1–5
My supervisor(s) supports the employees in the use of measurement instruments	84.3	4.41 (0.78)	1–5
My supervisor(s) use the measurement instruments in clinical practice themselves	86.0	4.10 (0.78)	1–5
My supervisor(s) requires employees to report digitally using the measurement instruments	88.6	4.20 (0.79)	1–5
My colleagues also use the measurement instruments in clinical practice	83.2	4.25 (0.63)	1–5
In our practice, the use of measurement instruments fits well in the way of working	88.0	4.15 (0.73)	1–5

^†^Since all items should have the same scoring procedure, this item was recoded positively

^‡^Score of 5 (totally agree) or 4 (agree)

^§^Scored on a five-point Likert scale 1 = totally disagree, 5 = totally agree

### Interviews

In total, 11 interviews were held. After discussing the preliminary results of 10 interviews, the researchers concluded that one more interview was needed to be certain that data saturation was reached. The interviews took between 30 and 70 min. Six of the interview participants were male (54.5%) with a mean age of 39.5 years, while the females (45.5%) had a mean age of 37.6 years. An overview of the characteristics of the participants is outlined in Additional file 3.

After analysing the data from the interviews, the codes were clustered into eight major categories: (1) Applicability and time frame of assessments of the measurement instruments in the standard set; (2) Knowledge and skills of physiotherapists; (3) Acceptance (including attitudes) of physiotherapists; (4) Patient motivation and behaviour; (5) Quality improvement; (6) Information system of the practice; (7) Availability of resources in the practice; (8) Transparency. These major categories were allocated to the seven generic domains identified by Flottorp et al. [21] (see Table 3). The categories are described in detail in the following paragraph.

**Table 3:** Categorization of the generic and specific domains, major categories, and codes

Generic domains according to Flottorp et al. [21]	Major categories	Codes
Guideline factors	Applicability and time frame of assessments of the measurement instruments in the standard set	Goals of using the measurement instruments
		Barriers to using the measurement instruments
		Facilitators of using the measurement instruments
		Presented information for using the standard set is sufficient
Individual health professional factors	Knowledge and skills of physiotherapists	Different experiences of using the measurement instruments related to additional COPD training
	Acceptance (including attitudes) of physiotherapists	Different experiences of using the measurement instruments related to age
		Mixed perspectives on different ways to use the data of the measurement instruments
Patient factors	Patient motivation and behaviour	Resistance to frequent measuring
		Interest in own results
		Participating in filling in questionnaires
		Enthusiasm towards using an accelerometer
		Barriers to using the accelerometer
Professional interactions	Quality improvement	Barriers to quality improvement
		Facilitators of quality improvement
		Barriers of having a small practice and little capacity
		Feedback on the measurement instruments results is valued
Incentives and resources	Information system of the practice	Barriers to the implementation
		Facilitators of the implementation
		Software problems
		Software facilitators
	Availability of resources in the practice	Lack of space to complete the 6MWT
		Microfet™ is expensive to purchase
		Shortage of accelerometers
Capacity for organizational change	Information system of the practice	Barriers to the implementation
		Facilitators of the implementation
		Software problems
		Software facilitators
	Availability of resources in the practice	Lack of space to complete the 6MWT
		Microfet™ is expensive to purchase
		Shortage of accelerometers
Social, political, and legal factors	Transparency	Positive perspectives towards making the anonymized standard set data transparent for policymakers
		Negative perspectives towards making the standard set data transparent at an individual level

*6MWT* six-minute walk test, *COPD* chronic obstructive pulmonary disease

#### Applicability and time frame of assessments of the measurement instruments in the standard set

Generally, the physiotherapists stated that the use of the measurement instruments in the standard set was feasible because they are sufficient to provide insight into the effect of the treatment without taking too much time to complete. The physiotherapists also indicated that the standard set was able to measure what is necessary to be able to evaluate and reorganize future treatment sessions based on the outcome, which is one of the goals of the use of the standard set:

“It [the standard set] guides your therapy and treatment plan and that of course has the effect that you have a better treatment plan for the patient and hopefully a better result” [I.09].

Despite the generally positive experience with the standard set, barriers were also identified for specific measurements. According to some physiotherapists, the Microfet™ was unreliable because it depends on the way in which it is used, as well as being affected by the experience of the physiotherapist:

“With the Microfet™, there is a difference in testing. There is too much of a difference in the outcomes between individuals [physiotherapists]. It is just very ‘sensitive’ to the way in which it is used” [I.10].

In general, the participants agreed that an accelerometer provides valuable insights into the general activity of the patient; however, some physiotherapists stated that the accelerometer is not accurate in estimating the number of steps per day:

“I always have doubts about the accuracy of the accelerometer, but it does give an indication […] Some patients still score very few steps, which gives me a good insight that I should speak to them to see how I can encourage them to move more. Otherwise, you would have no insight in that area” [I.06].

It was indicated in the instructions for the standard set that measurements should be conducted every 3 months; however, the physiotherapists stated that this was not always possible due to a lack of time or the status of the patient.

“[…] and sometimes patients have a bad day the day you were planning to measure the performance measures from the standard set, at those moments they are absolutely not motivated. Then it is difficult for me to tell them that we still need to perform the measures” [I.9]

During the interviews, variation was observed in the frequency at which the physiotherapists used the standard set; while some physiotherapists repeated the measurements on schedule, other physiotherapists reported using the measurement instruments every 6 months. In general, however, the standard set was found to be very useful. The participants commented that the standard set ensures that they measure consciously:

“Yes, I still use the standard set. With the standard set I learned to structurally measure outcomes. When it is really busy at work, and you think that you do not have time, then the standard set motivates me to measure the repeated measurement.” [I.10]

Generally, the physiotherapists thought that the information provided before and during the project was useful and easy to apply. The participants commented that the protocol was simple to implement and follow.

Barriers associated with the instructions were also mentioned during the interviews, however. Some physiotherapists indicated that when specific measurement instruments are not available (e.g., the Microfet™) or cannot be performed exactly according to the instructions (e.g., no ten-metre space available for the 6MWT), alternative measurement instruments or instructions should be given. Moreover, according to the physiotherapists, the fact that some measurement instruments are optional should be made clearer in the information provided for the standard set:

“I understood that [some instruments are optional], but colleagues of mine asked: ‘we should also take that test, right?’ ‘Well, that is not necessary with this client because that is not a goal. His strength is already good, so you don’t need to test that further’. It was in the text [information for the standard set], but maybe mention it more often in several places or something” [I.01].

#### Knowledge and skills of physiotherapists

The interviews revealed that physiotherapists who had not received additional COPD-specific training had less of a positive experience with the use of the standard set of measurement instruments because they lacked the underlying knowledge of these procedures. According to the participants, however, skills are related to the experience of the physiotherapists. The participants also stated that specialized physiotherapists should continuously develop their knowledge by undertaking additional training to keep themselves more alert about their clinical process:

“I always find that when I have completed a COPD training course, I am more up-to-date and alert. But I think that applies to everyone” [I.07].

#### Acceptance (including attitudes) of physiotherapists

The participants indicated that the younger generation of physiotherapists are trained in using measurement instruments and reporting the data, and therefore have more positive experiences of using them than the older generation. The critical attitudes of older physiotherapists were noted as a barrier for using measurement instruments, as this group is less familiar with them. The physiotherapists indicated that the use of measurement instruments comes with too much reporting, which is time consuming:

“I know a lot of colleagues in my age group who think that it is all nonsense [the use of measurement instruments] and do not want to explore the use of measurement instruments and start working with them. And yes, that is a pity” [I.04].

#### Patient motivation and behaviour

The physiotherapists mentioned that the motivation of the patient is built on providing sufficient information about the importance of using measurement instruments. The participants indicated that some patients were not motivated to complete the measurements every 3 months because they are not used to routine testing; however, most of the patients were interested in their results and were therefore more motivated to complete the standard set of measurements:

“What is striking is that the patients also like to evaluate the results every three to four months, to do all the tests and measurements. They are also interested to see how they are doing, not only in the function of their lung” [I.06].

The physiotherapists mentioned that the limited number of questionnaires included in the standard set meant the patients had no problems completing them. The ability to complete the questionnaires online also motivated the patients because it takes less time. The use of instruments that allow patients to track their activity made them more keen to complete the measurements and tests. According to the participants, this was because a goal (amount of steps) was given to the patients:

“I do see, when the patients get such a goal, that they like it. They say things like: ‘oh, I’ve taken 5000 steps, let’s try to set 5500 or so this week’” [I.02].

#### Quality improvement

Both barriers and facilitators were mentioned for using aggregated outcomes for quality improvement purposes by comparing outcomes between physiotherapists. Although this goal is valued, the physiotherapists experience the use of the data as confrontational because the scores are compared between physiotherapists, and some have higher scores than others:

“It is very confrontational for the treating physiotherapists and they defend themselves. I myself also tend to do it, because you sometimes feel more or less attacked. It shouldn’t be like that; it has to be for learning, it has to be for improvement” [I.07].

The physiotherapists indicated that the results of the measurement instruments should be case-mix corrected for the burden of COPD, because this condition explains the results to a large extent. This will facilitate the use of the data for quality improvement:

“If, for example, one physiotherapist treats more patients in classes A and B [burden of disease] and the other more from C and D, what do these data say then?” [I.12].

The physiotherapists stated that the use of data for quality improvement initiatives also depends on the size and capacity of the practice. Most small practices employ less specialized physiotherapists, who therefore receive less feedback from other physiotherapists with the same specialism and have fewer colleagues with whom to compare their data. The participants who were the only COPD-specialized physiotherapist of their practice indicated that they were curious about their own outcomes and willing to compare outcomes with colleagues:

“We can use the graphs to see whether there is a difference [between the scores of physiotherapists], and then we can explore where that difference comes from. I believe in that way we can learn from each other” [I.09].

To facilitate the use of the data from the measurement instruments for quality improvement, it is useful for the physiotherapists to be able to record specific factors, such as changes in medication or hospitalization. In that way, the cause of the possible variation between physiotherapists within a practice could be identified more easily.

The visual feedback of the results of the standard set in caterpillar plots was highly valued by the participants, who felt they could easily use the data to compare their results with those of other physiotherapists.

Moreover, the physiotherapists enthusiastically indicated that they want to use the data in their practice to improve their quality. As the data would be anonymously provided, they indicated that practices must be able to contact other practices with better scores to be able to learn how to improve their own quality without violating the privacy of other practices.

A major barrier for using the feedback of the results is that some physiotherapists received feedback with less data than they had sent. The physiotherapists indicated that they would therefore have appreciated receiving more feedback then twice a year a feedback report comparing their own collected data with benchmark data report. For example, the appreciated to be informed when their data has been received:

“These data would be nice to present between the feedback moments. You [the person who receives the data] could ask after six months or a year: ‘we now have received this number of measurements. Is this in line with the number of patients you treat and for whom you have taken the measurements?’ If it is not correct, then you can try to find the reason behind it” [I.06].

#### Information system of the practice

Most physiotherapists indicated that they did not experience problems with the way the standard set was implemented, nor with the software they were using:

“A protocol for the standard set was just assigned, right? So, we could actually just implement that” [I.09].

Other physiotherapists mentioned several missed opportunities concerning the implementation of the protocol of the standard set and the software; for example, some participants indicated that their practice found it difficult to correctly implement the standard set at the beginning of the study. Most of the physiotherapists mentioned that this was due to the way their practice leader or colleague had informed them about how to find the standard set in their electronic health record:

“We were not informed correctly, as that colleague [who informed the others about the standard set] actually started that trajectory before fully implementing the standard set in our system. They just told us what the intention was and how we should start with it” [I.05].

#### Availability of resources in the practice

Most participants commented that all measurement instruments, and the resources required to properly use them, were available; however, a few participants stated that some measurement instruments were not available in their practice. This was mainly true for the Microfet™, a tool to measure muscle strength, as this instrument is expensive to purchase:

“So the hand-held dynamometer, we don’t have that in our practice. As an investment it is quite expensive. So, eventually we never decided to buy the Microfet™” [I.08]

Also, some of the participants mentioned that some practices do not have enough space to optimally use some of the measurement instruments. This specifically holds true for the six-minute walk test (6MWT), for which it is advised that the patients walk ten metres back and forth in a straight line, but this is not possible in every practice.

#### Transparency

Another aim of the project was to make the anonymous results of the measurement instruments transparent for stakeholders and eventually to make the data totally transparent on physiotherapist or practice level. All physiotherapists indicated that the transparency of the data is an important factor for improving the quality of care; however, the participants indicated that the data collection should be optimized before it is used for external transparency purposes. All physiotherapists stated that it is important to perform a case-mix correction for the burden of COPD. Furthermore, the physiotherapists indicated that it is important to harmonize the use of the measurement instruments:

“I think it is good to compare between different practices, but it is not enough with the measurements we use now because, for example, the six-minute walk test can be measured in many different ways” [I.09].

Most of the physiotherapists indicated having no problem with the data being accessible in an anonymous form for policymakers when it is case-mix corrected, as mentioned above:

“I think that the more information we can provide to policymakers, the better the directives they write” [I.06].

The physiotherapists mentioned that making the data of the measurement instruments totally transparent on an individual level is important, yet they expressed some concerns related to potential gaming because they fear the negative (financial) consequences. A third party would therefore be needed to perform the measurements, according to some participants. Another barrier mentioned was that the physiotherapists think that both patients and health insurers might misinterpret the results:

“It is always difficult to know how another party would interpret such data. You may want to be transparent because it is important to you, but I am not sure that the patient who reads it can interpret it correctly” [I.10].

## Discussion

The results of our study show that the participating physiotherapists in the survey had a positive attitude towards, felt knowledgeable about, and were supported by practice policy and colleagues in the use of a standard set of measurement instruments with patients with COPD. The qualitative analysis resulted in experiences of physiotherapists with implementing the standard set of measurement instruments into eight defined categories. Although some barriers were mentioned, the physiotherapists during the interviews valued using the measurement instruments on patient-level for the evaluation of physiotherapy treatments, as well as on aggregated-level for quality improvement purposes. Moreover, the physiotherapists indicated that the measurement instruments have added value for the anonymized publication of outcomes, providing transparency to policymakers. To our knowledge, this was the first study that used a mixed methods design to evaluate the experiences of physiotherapists regarding the implementation of a standard set of measurement instruments for the improvement of primary care physiotherapy treatments for patients with COPD.

In accordance with the present evaluation of the implementation of a standard set, previous studies have identified barriers for implementing a guideline for COPD physiotherapy treatment [22]. Similarities were found in both the positive attitude of physiotherapists towards using measurement instruments and the negative finding that using measurement instruments takes too much time [22]. In more general studies of the use of measurement instruments in physiotherapy, lack of time was again reported as a barrier [9, 23–25].

Another important finding was that patients are more motivated to undertake the tests when sufficient information about the importance of the measurement instruments is provided by the physiotherapists. This is consistent with the study of Østergaard et al*.* [26], in which the patients were found to be less active when physiotherapists did not provide information about the importance of physical activity.

To identify the experiences of primary care physiotherapists regarding the use of measurement instruments, most researchers only used surveys and focussed on specific outcome instruments [23, 27]. The present study used a survey combined with semi-structured interviews to provide additional information and explanations to the answers given in the survey. Moreover, this design was used to create a complete overview of the experience of using all the measurement instruments, including the use of the data for quality improvement and transparency.

Prior studies have also developed standard or core sets of outcome measures for patients with COPD [28–32]. Most of these sets were to be used in clinical trials [28, 29, 31] or were not designed for the evaluation of the physiotherapy treatment of patients with COPD [30, 32]. In the current study, we evaluated the implementation of the standard set that was developed for use in Dutch primary physiotherapy care. We believe that researchers, policymakers, and other stakeholders can learn from the experiences of physiotherapists using the standard set of measurements to collect aggregated outcomes for quality improvement and external transparency.

### Limitations

This study has several limitations. First, interviews were held by only one of the researchers (JZ) and member checking was not performed. This could have negatively influenced the trustworthiness and validity of this study [33]. To strengthen trustworthiness, the interviews were independently analysed, and codes were assigned by JZ and AV.

Second, as only physiotherapists were interviewed in this study, it is important to indicate that the barriers and facilitators allocated to the domain ‘patient factors’ were based on the perception of the physiotherapists and not obtained from the patients themselves.

Lastly, the major categories that emerged in this study were allocated to the seven domains developed by Flottorp et al*.* [21]; however, the domains ‘incentives and resources’ and ‘capacity for organizational change’ both contained the same two major categories because no distinction could be made when allocating the different codes and categories to the domains. Despite this issue, data saturation was obtained and a consensus about the findings was reached as all research members discussed the codes, categories, and domains several times during the study.

### Implications for practice

Feedback regarding the outcome data might promote quality improvement, but its effectiveness is related to how the feedback is provided [34]. The physiotherapists indicated that the feedback is very useful for quality improvement; thus, it can be assumed that feedback regarding the measurement instruments can contribute to quality improvement initiatives. This could lead to better physiotherapy treatment for patients with COPD; however, future research should explore how the use of measurement instruments affects the quality of physiotherapy treatment and the outcomes of care.

The physiotherapists indicated that they were sceptical and not prepared to provide their data for full transparency yet, because they believed it likely that other physiotherapists would manipulate their outcomes to avoid negatively affecting their reimbursement by health insurers. The participants therefore suggested that the measurements should be performed by a third party. This idea should be explored in the future before making the outcomes totally transparent.

Sets of measurement instruments are always subject to change, and the routine evaluation of the instruments is always necessary. We will therefore routinely discuss, improve, implement, and evaluate the standard set in future research.

## Conclusion

This mixed method study shows that the participating physiotherapists supported the use of a standard set of measurement instruments to improve the quality of physiotherapy treatment for patients with COPD. Eight categories were identified in the physiotherapist experiences with the use of the standard set for these patients. Moreover, we showed that the routine use of the set of measurement instruments has the potential to be used for the anonymized publication of outcome data, providing transparency to policymakers. The results of this study could be used for future projects focussed on improving, implementing, and evaluating the standard set.

## Supplementary Information

**Additional file 1:** Overview of the standard set of measurement instruments [13].

**Additional file 2:** Interview guide.

**Additional file 3:** Characteristics of the physiotherapists participating in the individual interviews.

## Data Availability

The data that support the findings of this study are available from the corresponding author, AV, upon reasonable request.
