# Supplementary material for: Experiences of physiotherapists regarding a standard set of measurement instruments to improve quality of care for patients with chronic obstructive pulmonary disease: a mixed methods study
Source: J Patient Rep Outcomes. 2022 Jul 19;6:79. doi: 10.1186/s41687-022-00487-2 (PMC9296726; doi:10.1186/s41687-022-00487-2)
Supplement: Supplementary file 1 — Additional file 1: Overview of the standard set of measurement instruments [13]. [file 41687_2022_487_MOESM1_ESM.pdf]

Supplementary File 1 | Overview of the standard set of measurement instruments [13].

nr	Domain	Measure	Guided measurement protocol		
		**A: mandatory for all patients with COPD**	**Intake**	**Every three months**	**End of treatment**
1	Practice/physical therapist level	Characteristics of practices and physical therapists	Once a year		
2	Physical capacity	Six-minute walk test (6MWT)	X	X	X
3	Quality of life	Clinical COPD Questionnaire (CCQ)	X	X	X [a]
4	Experience	Global Perceived Effect (GPE)		X	X
		**B: Conditional measures**			
5	Muscle strength	hand-held dynamometer (HHD) (with a Microfet™) for quadriceps strength	X	X	X
6	Dyspnoea	Medical Research Council Dyspnea (MRC)	X	X	X
		**C: Exploratory measures**			
7	Physical activity*	Accelerometer (for physical activity in daily life)			
8	Assessment of Burden of COPD tool (ABC) tool*	The Assessment of Burden of COPD tool			
		**D: Classifying subgroups**			
9	Classify in subgroups	Classify subgroups based on the Dutch care standard of the Lung Alliance.[34]	Once a year		

Note: [a] After ≥12 months the CCQ needs only to be measured once a year.

The two exploratory measures were used in a separate pilot in a small subgroup, the measurement protocol was developed in that pilot
