# Supplementary material for: Experiences of physiotherapists regarding a standard set of measurement instruments to improve quality of care for patients with chronic obstructive pulmonary disease: a mixed methods study
Source: J Patient Rep Outcomes. 2022 Jul 19;6:79. doi: 10.1186/s41687-022-00487-2 (PMC9296726; doi:10.1186/s41687-022-00487-2)
Supplement: Supplementary file 2 — Additional file 2: Interview guide. [file 41687_2022_487_MOESM2_ESM.pdf]

Supplementary File 2 Interview guide

Introduction and informed consent

1. What do you think of how well the steps enabled you to use the standard set?
2. What do you think was the goal of the standard set project?
3. Did your practice leader clearly inform you about the standard set project? If not, what could have been done better?
4. In what way do you use the standard set in your practice?

Introduction to graphs of the data from the standard set for quality improvement

5. What do you think about the use of the data from the standard set for quality improvement?
6. Do you compare the results of different physiotherapists in your practice when using the standard set? If yes, how do you do that? Do you learn from this comparison? In what way does that add value to the quality of care? If not, why do you not compare them? Please state whether you would like to work like this in the future and why.

Introduction to graphs of the data from the standard set for transparency

7. Do you think that the use of the anonymized data from the standard set has added value for policymakers?
8. Do you see any barriers to this anonymized use?
9. What do you think of making the results completely transparent but in a non-anonymous way, for example for patients?
10. Do you think the use described in question 9 would have added value?
11. Do you see any barriers to the use of data described in question 9?
12. How did the delivery of your data go? Did you experience any problems?
13. How was the feedback you received regarding the data? Did you experience any problems?
14. What did you think of the feedback report?
15. How do you think the measurement instruments contribute to improving physiotherapy for patients with COPD?
16. In your opinion, what could be a reason for not using the measurement instruments in physiotherapy treatments for patients with COPD?
17. What points do you think should be improved in the standard set in order to be able to fully apply it in daily practice?
18. What do you think of your own knowledge and skills regarding the use of the measurement instruments?
19. What could be a disadvantage of using the measurement instruments?
20. Are there other strengths and weaknesses of the standard set that you want to mention?
21. How do you ensure you have enough knowledge regarding the treatment of patients with COPD? (techniques, colleagues, literature, additional COPD training, etc.)
22. What COPD-specific training have you undertaken OR what is the reason you did not undertake additional COPD training?

General questions: age, years of physiotherapeutic experience, specialism, size of the practice.
