# Supplementary material for: Experiences of physiotherapists regarding a standard set of measurement instruments to improve quality of care for patients with chronic obstructive pulmonary disease: a mixed methods study
Source: J Patient Rep Outcomes. 2022 Jul 19;6:79. doi: 10.1186/s41687-022-00487-2 (PMC9296726; doi:10.1186/s41687-022-00487-2)
Supplement: Supplementary file 3 — Additional file 3: Characteristics of the physiotherapists participating in the individual interviews. [file 41687_2022_487_MOESM3_ESM.pdf]

Supplementary File 3 | Characteristics of the physiotherapists participating in the individual interviews.

ID	Gender	Age	Working hours per week	Experience in years	Additional COPD-training
I.01	Female	35	32	10	Yes
I.02	Female	27	32	7	Yes
I.03	Male	33	36	11	Yes
I.04	Male	60	30	35	No
I.05	Female	38	20	16	Yes
I.06	Male	38	30	14	Yes
I.07	Male	58	25	36	Yes
I.08	Female	44	22	17	Yes
I.09	Male	24	37	2	Yes
I.10	Female	44	27	23	Yes
I.11	Male	24	36	2	Yes
